# Molecular Characterization of Reactive Oxygen Species in Myocardial Ischemia-Reperfusion Injury

**DOI:** 10.1155/2015/864946

**Published:** 2015-10-05

**Authors:** Tingyang Zhou, Chia-Chen Chuang, Li Zuo

**Affiliations:** ^1^Radiologic Sciences and Respiratory Therapy Division, School of Health and Rehabilitation Sciences, The Ohio State University College of Medicine, The Ohio State University Wexner Medical Center, Columbus, OH 43210, USA; ^2^The Interdisciplinary Biophysics Graduate Program, The Ohio State University, Columbus, OH 43210, USA

## Abstract

Myocardial ischemia-reperfusion (I/R) injury is experienced by individuals suffering from cardiovascular diseases such as coronary heart diseases and subsequently undergoing reperfusion treatments in order to manage the conditions. The occlusion of blood flow to the tissue, termed ischemia, can be especially detrimental to the heart due to its high energy demand. Several cellular alterations have been observed upon the onset of ischemia. The danger created by cardiac ischemia is somewhat paradoxical in that a return of blood to the tissue can result in further damage. Reactive oxygen species (ROS) have been studied intensively to reveal their role in myocardial I/R injury. Under normal conditions, ROS function as a mediator in many cell signaling pathways. However, stressful environments significantly induce the generation of ROS which causes the level to exceed body's antioxidant defense system. Such altered redox homeostasis is implicated in myocardial I/R injury. Despite the detrimental effects from ROS, low levels of ROS have been shown to exert a protective effect in the ischemic preconditioning. In this review, we will summarize the detrimental role of ROS in myocardial I/R injury, the protective mechanism induced by ROS, and potential treatments for ROS-related myocardial injury.

## 1. Introduction

Myocardial ischemia-reperfusion (I/R) injury occurs when the blood flow to the myocardium is obstructed, followed by the restoration of blood to the ischemic heart [1, 2]. Ischemia to a specific region of the body can provoke tissue damage due to lack of oxygen and nutrients; heart is particularly vulnerable since it demands high energy to function [1, 3]. It is reasonable to consider that the rapid and early restoration of blood flow to the ischemic regions prevents further damage. However, numerous studies have observed the reduced cardiac function and even the acceleration of myocardial injury after reperfusion [1, 3, 4]. Ischemic injury is a recognizable consequence of cardiovascular diseases including myocardial infarction, stroke, and coronary heart diseases. Moreover, some patients who suffer from severe coronary heart disease choose to undergo a coronary artery bypass grafting to promote cardiac function, yet they experience myocardial injury postoperatively [3, 5].

In response to a sudden ischemia, coronary vessels dilate to compensate for the low oxygen supply, allowing for maximal oxygen return/recirculation [6]. However, the continuous deficiency of oxygen during ischemia shifts cardiac metabolism toward anaerobic glycolysis, disrupts ATP generation in the mitochondrial oxidative phosphorylation (accounting for 95% of ATP production in the heart), and thus reduces overall ATP availability [3]. The ATP-dependent ion pumps, such as sodium potassium (Na^+^/K^+^) ATPase and calcium (Ca^2+^) ATPase, are disturbed largely by the ATP depletion. Secondary channels, including Na^+^/H^+^ exchanger, and decreased intracellular pH, lead to intracellular Na^+^ and Ca^2+^ overload [3, 6, 7]. Subsequently, the altered ion homeostasis and metabolism reduce cardiac contractility and structural organization, and initiate cell death via necrosis and apoptosis [3, 6]. Replenishment of blood during reperfusion rapidly restores cellular balance in the myocardium and therefore prevents further ischemic injury.

Perhaps somewhat counterintuitively, such normalization concurrently causes injury [6]. The molecular mechanisms regarding I/R injury are multifactorial, and various hypotheses have been proposed to describe the pathogenesis of myocardial I/R [7, 8]. Reperfusion treatments, including thrombolysis and percutaneous coronary interventions, serve to manage the ischemic conditions but unexpectedly induce additional damage [9, 10]. Indeed, the reestablishment of blood flow may trigger apoptosis and necrosis in the myocardium [8].

A growing number of studies have investigated how reactive oxygen species (ROS) play an intriguing role in I/R, ranging from beneficial to inimical [7, 11]. At the basal level, ROS function as a mediator for multiple cellular signaling cascades including cell growth and stress adaptation [9]. Conversely, excess ROS can damage tissues by oxidizing important cellular components such as proteins, lipids, and DNA, as well as activating proteolytic enzymes such as matrix metalloproteinases [7, 12]. Furthermore, the induction of protective mechanisms during ischemic preconditioning (IPC) can be associated with low level of ROS [7, 11]. The production of ROS has been implicated in both myocardial ischemia and reperfusion injury. During the past decade, studies continued to explore the mechanisms as well as develop clinical applications centered on ROS-related therapies to alleviate I/R injuries. More recently, novel concepts and treatments such as postconditioning and mesenchymal stem cells-based interventions have been thoroughly updated focusing on ROS-centered interventions in myocardial I/R diseases [13–16]. By highlighting these innovative findings, the current paper provides a timely review of the latest understanding of I/R and associated protective mechanisms linked to the unique role of ROS.

## 2. Involvement of ROS during Myocardial Ischemia

### 2.1. Physiological Relevance of ROS in the Cardiovascular System

ROS are essential in mediating physiological responses [12, 17]. Upon the exposure to environmental stresses such as hypoxia, ROS production can be significantly elevated to a level that overwhelms the endogenous antioxidant system and engenders tissue damage [7, 12, 17]. Typically, a small amount of superoxide (O_2_^∙−^, a type of ROS) is generated through electron leakage in mitochondrial electron transport chain, which can further lead to the formation of other ROS such as hydrogen peroxide (H_2_O_2_) and hydroxyl radical (^∙^OH) [3, 9, 18]. These byproducts of respiration may exert beneficial effects on cardiovascular functions and can be safely metabolized by the antioxidants under normal conditions [19, 20]. Cardiac mitochondria have been recognized as an important source of ROS in the myocardium, considering that a large number of mitochondria reside in the cardiomyocytes to meet a high energy demand [3, 20]. NADPH oxidases (Nox) also contribute to the major production of O_2_^∙−^ and H_2_O_2_ in cardiovascular cell types. Particularly, highly expressed Nox2 and Nox4 isoforms in the heart play an essential role in regulating the development of cardiomyocytes [21–23]. Other O_2_^∙−^ generating systems include lipoxygenase and xanthine oxidase (XO) [9, 18]. Moreover, the predominant expression of  XO under stress has been shown to contribute to ROS generation in the perfused ischemic tissue [9].

### 2.2. ROS Formation during Ischemia

Although reperfusion is responsible for generating a ROS burst during reintroduction of molecular oxygen (O_2_) to the ischemic environment, accumulating evidence has suggested that oxidant stress commences before reperfusion [11, 17]. Indeed, Zhu and Zuo observed a rapid increase in O_2_^∙−^ production within three minutes of ischemia [17]. Interestingly, the study identified oxymyoglobin as a novel source of O_2_^∙−^ generation in the rodent heart model during early ischemia, through an interaction between the iron ion in the heme group and reduced oxygen tension [17]. Myoglobin can also serve to reservoir O_2_ but is quickly exhausted [3].

**Figure 1:**
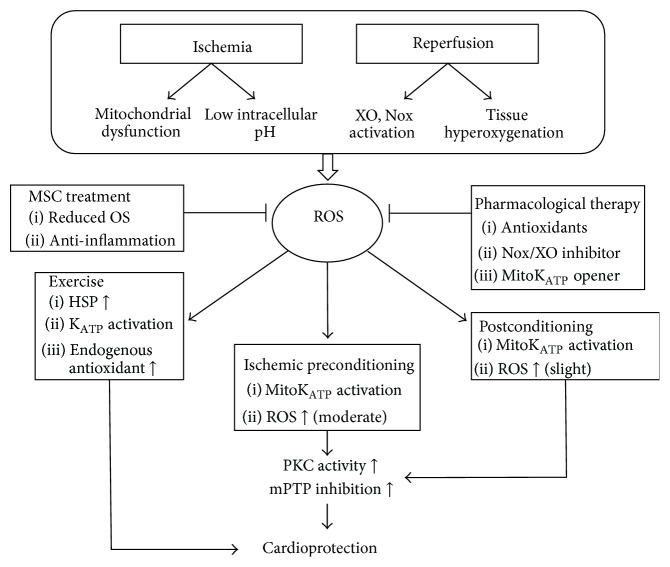
This schematic summarizes the role of reactive oxygen species in myocardial ischemia-reperfusion and related treatments. Reactive oxygen species generated during myocardial ischemia-reperfusion are involved in multiple cellular pathways that eventually lead to cardioprotection. HSP: heat shock protein; K_ATP_: ATP-sensitive K^+^ channel; mitoK_ATP_: mitochondrial ATP-sensitive K^+^ channel; mPTP: mitochondrial permeability transition pore; MSC: mesenchymal stem cell; Nox: NADPH oxidase; OS: oxidative stress; PKC: protein kinase C; ROS: reactive oxygen species; and XO: xanthine oxidase.

Studies have noted that the presence of residual O_2_ is a critical element for ROS generation during ischemia, in which an impaired mitochondrial electron transport chain is believed to facilitate the conversion of residual O_2_ to O_2_^∙−^ due to increased electron leakage (Figure 1) [11, 20]. Levraut et al. detected an irreversible decline in mitochondrial membrane potential in ischemic chicken cardiomyocytes. ROS-induced mitochondrial depolarization during ischemia is associated with myocyte death in I/R; specifically, the severity of depolarization is related to the extent of cell death in perfused tissues [11, 24]. The decline in membrane potential may be attributed to the opening of mitochondrial permeability transition pore (mPTP). However, the inhibition of mPTP activation does not significantly prevent depolarization, whereas the application of antioxidants restores membrane potential and prevents subsequent cell death [11]. In fact, mPTP opening is limited during ischemia due to low pH environment, but mPTP does play a crucial role in reperfusion injury [25]. At reperfusion, mPTP is activated, which exacerbates injury via ROS-induced ROS release cycle and initiates cell death signaling pathways [7]. Collectively, Levraut et al. proposed a putative scheme of ischemic injury involving the disruption of mitochondrial inner membrane by ROS-induced lipid peroxidation that contributes to the repolarization failure in mitochondria later during reperfusion [11]. Moreover, the impediment of mitochondrial depolarization and Ca^2+^ dysregulation by sarcolemma stabilizer suppress apoptotic and necrotic pathways, despite the presence of oxidative stress (OS) and an altered redox state [24].

In an attempt to detoxify the oxidative insult, a significant rise in the antioxidant defense system, such as glutathione increment, has been observed during ischemia [4]. In addition, the formation of O_2_^∙−^ at the early period of ischemia is likely to be involved in facilitating protections towards ischemic tissues, which is the so-called IPC [15, 17]. A detailed discussion of this will be provided later in the review.

## 3. ROS Mechanism in Myocardial I/R

### 3.1. A Burst of Oxidants during Reperfusion

It is true that a timely reperfusion is essential to ease ischemic injury and salvage viable myocardium, yet it can promote cardiomyocyte damage [26]. During reperfusion, the reactivation of aerobic metabolism induces an increase in ROS, particularly O_2_^∙−^, which exceeds the endogenous antioxidant capacity [3, 6]. The shift towards a more reduced redox state during ischemia may also contribute to the production of ROS upon reoxygenation [4]. The reduced form of iron ion mediates the Fenton chemistry to generate highly reactive hydroxyl ions [27]. Of various cellular alterations in the perfused ischemic myocardium, distorted redox state is widely recognized in the initiation of I/R cellular damage, although the precise role of ROS in I/R remains elusive [24].

The overproduction of O_2_^∙−^ derives further ROS generation in the mitochondria, forming a vicious cycle of OS [20, 27]. For instance, the reaction between aconitase (a mitochondrial protein) and O_2_^∙−^ leads to the formation of ^*∙*^OH [20]. The aconitase inactivation can be therefore regarded as an indicator for O_2_^∙−^ toxicity [27]. An activation of XO during reperfusion is attributed not only to oxygen influx, but also to excess hypoxanthine (a substrate of XO) that is produced during ischemia as a result of ATP degradation [3, 10, 17]. In addition, ROS mediate the infiltration of neutrophils, which contribute to further O_2_^∙−^ generation via Nox activation (Figure 1) [26]. Increased OS during I/R results in the uncoupling of nitric oxide synthase (NOS), which reduces NO production and enhances NOS-derived O_2_^∙−^ level [28]. Those studies attributing the myocardial injuries to the decreased NO production during reperfusion are primarily based on the fact that, by applying NO or NO donors prior to I/R, the related injuries can be remarkably attenuated [29–33]. For instance, Liu et al. have proposed that the reperfusion-induced endothelial dysfunction can be caused by the decrease of NO formation, resulting in the diapedesis and neutrophil adherence in the ischemic region, thereby exacerbating I/R injuries [34]. Moreover, NO can react with excessive O_2_^∙−^ to form peroxynitrite (ONOO^−^), an oxidant that suppresses the mitochondrial respiration by modulating the nitration of complexes I and IV [4, 35]. Since the respiratory oxygen consumption is interfered, additional ROS may be generated due to tissue hyperoxygenation (Figure 1) [4, 36].

### 3.2. Detrimental Effects Induced by ROS

As discussed earlier, overexuberant ROS that overwhelm the antioxidant defenses can induce protein denaturation and cause oxidative damage directly to DNA, especially mitochondrial DNA due to its proximity to the dysfunctional electron transport chain [26, 37]. The destabilization of mitochondrial and sarcolemmal membranes caused by lipid peroxidation enables the influx of nonspecific, small molecules across the membrane, resulting in mitochondrial matrix swelling and apoptosis [6, 11, 24]. ROS trigger inflammatory cascades and expressions of adhesive molecules, leading to leukocyte/capillary plugging and endothelial swelling that interfere with capillary flow. Matrix metalloproteinases and other proteases are also activated by ROS, further deteriorating the functions of multiple proteins such as glycolytic and antioxidant enzymes [7, 38]. Impaired ROS and Ca^2+^ regulation can propagate to spread the injury through gap junctions [6]. Furthermore, the molecular alterations at the onset of reperfusion, such as ROS burst, recovery of pH, and Ca^2+^ overload, all facilitate the abrupt opening of mPTP, which is critical to reperfusion injury [7, 25, 39]. Although transient mPTP opening is involved in cardioprotection, prolonged opening results in irreversible changes within cellular bioenergetics and cell death due to the release of proapoptotic factors (e.g., cytochrome *c*) [7, 25]. Mitochondrial cardiolipin peroxidation also liberates cytochrome *c* and enhances electron leakage at complexes I–III [3, 7]. The administration of mPTP inhibitor has been reported to reduce myocardial infarct size in animal models, serving as promising therapeutic to avoid lethal myocardial reperfusion injury [26]. Despite the numerous detrimental outcomes related to ROS, repair processes including vascular remodeling and angiogenesis may occur at the later stage of reperfusion when ROS production returns to lower levels and resumes their roles as signaling molecules [7, 40].

### 3.3. Clinical Outcomes of Reperfusion Injury

Myocardial stunning is one of the clinical consequences associated with reperfusion injury. Although cellular homeostasis has been reestablished after reperfusion, the reversible contractile dysfunction may remain persistent and it is likely attributed to multiple mechanisms such as decreased ATP resynthesis [10, 26]. The sudden changes in ion concentration after reperfusion in the effort to restore balances induce reperfusion arrhythmias, a condition frequently experienced by patients undergoing surgical revascularization [10]. Reperfusion arrhythmia, such as ventricular fibrillation and ventricular tachycardia, is primarily responsible for the sudden cause of death after blood flow restoration [10, 41]. Moreover, microvascular obstruction is observed after reperfusion in which the blood cannot completely reperfuse to the ischemic region after the release of occlusion [10, 41]. Based on the prior discussions, it is likely that ROS play an important role in these events and further investigation is necessary.

## 4. Protective Role of ROS in IPC

Although excessive ROS production has been indicated as a primary contributor of I/R injury, ROS-targeted therapies including antioxidant treatments have yielded mixed results in attenuating I/R-induced damage [19]. The accumulation of such evidence suggests the potential role of ROS in the protection of cardiomyocytes. Indeed, studies have shown that the application of antioxidants impedes preconditioning protection. ROS formation during both ischemia and reperfusion is likely to participate in the protective mechanisms induced by preconditioning. Particularly, mitochondrial ROS are paramount in signaling IPC as detailed below [19].

### 4.1. Myocardial Preconditioning

Murry et al. first described the protective effect of preconditioning on myocardium in 1986 and observed a slower ATP depletion rate and a smaller infarct size in the heart treated with brief episodes of I/R cycles before prolonged occlusion followed by reperfusion [42, 43]. Later research recognized several types of preconditioning protocols including IPC, exercise preconditioning, and pharmacological preconditioning [2, 44, 45]. Preconditioning provides a beneficial “warm-up” that primes the tissue to subsequent injuries when it is subjected to prolonged stresses such as ischemia and hypoxia [2, 46]. Specifically, IPC is marked by transient exposures of sublethal I/R that induces myocardial protection against later I/R injury. Small amounts of ROS generated during short periods/cycles of I/R are indeed highly associated with the protective effect exerted by preconditioning [2]. In particular, ROS originating from the mitochondria play a pivotal role in mediating cardioprotection via mechanisms involving the activation of survival programs [2, 7]. Moreover, lower OS has been observed in I/R preconditioned cardiac muscle during prolonged I/R, which is attributed to the reduced ROS generation in mitochondria [2]. One of the well-established IPC mechanisms involves the opening of mitochondrial ATP-sensitive K^+^ (mitoK_ATP_) channel (Figure 1). MitoK_ATP_ channel is activated upon the exposure to preconditioning stimuli while the subsequent influx of K^+^ leads to depolarization and matrix alkalization, which consequently induces a moderate increase in ROS and the activation of downstream survival signaling events [7, 47]. Notably, these preconditioning-induced ROS may mediate protein kinase C (PKC) activity and, via multiple steps, inhibit the opening of mPTP (Figure 1) [2, 7]. As discussed earlier, mPTP is a major regulator of necrosis and apoptosis [48]; such inhibition of mPTP opening is therefore essential to the cardioprotection [2]. In addition, the opening of the mitoK_ATP_ channel can generate mild matrix swelling which can improve ATP synthesis and fatty acid oxidation, leading to cardioprotective effects [2]. The application of mitoK_ATP_ openers mimics IPC whereas K_ATP_ blockers, such as 5-hydroxydecanoate, attenuate cardioprotection, further suggesting the importance of mitoK_ATP_ in IPC protection [47]. Besides the exclusive role of mitochondrial ROS in signaling IPC [7], the initial burst of ROS is correlated with IPC efficacy, and it serves as essential preconditioning stimulus to the activation of mitoK_ATP_ as well as sarcolemmal K_ATP_ (sarcK_ATP_) [2, 49].

Cardioprotection can also be provided by preconditioning of regular or mild exercise [2]. Exercise has long been known for its potential to prevent cardiovascular diseases by modulating related risk factors such as obesity and hypertension [45]. Termed exercise preconditioning, exercise-induced protection involves the interplay of several protective mediators including endogenous antioxidants that eventually leads to myocardial-specific biochemical adaptations [45, 50]. For instance, exercise stimulates the upregulation of heat shock proteins (HSPs), a group of proteins that is overexpressed spontaneously under stressful events (Figure 1) [50]. HSPs are equipped by the cell as a defense mechanism in order to maintain cellular homeostasis. Specifically, HSPs are responsible for assisting proper folding of proteins and facilitating degradation of damaged proteins [50]. The increased HSP activity may certainly be beneficial in the protection against I/R injury [51]. Despite the cardioprotection exerted by HSPs, studies have shown that the application of antioxidants on exercised animal can suppress HSP72 expression, yet the cardioprotective effects remain unaffected [52]. However, several studies have reported that the cardioprotective effect exerted by exercise against reperfusion injuries can be abolished when antioxidants are administrated during exercise, indicating the crucial role of ROS signaling in exercise-induced preconditioning pathways [53–55]. Similar to IPC, the activation of mitoK_ATP_ and sarcK_ATP_ channels in the exercised heart, where elevated PKC level is evidenced, also contributes significantly to cardioprotection (Figure 1). However, the exact role of K_ATP_ channels in exercise preconditioning remains ambiguous [45, 56]. Most importantly, exercise enhances endogenous antioxidant systems and improves ATP synthesis and mitochondria functions, which all serve to strengthen and increase tolerance of heart [51].

### 4.2. Postconditioning

Ischemic postconditioning, first put up by Zhao et al. in 2003, demonstrates a cardioprotection that is tantamount to IPC in a nonpretreated heart after I/R [13]. It is later defined as “brief periods of ischemia alternating with brief periods of reflow applied at the onset of reperfusion following sustained ischemia” [13, 57]. Since reperfusion injuries occur within several minutes of blood reflow, postconditioning must be introduced as soon as the reperfusion is initiated [57]. Basically, postconditioning and preconditioning follow similar protocol in which the myocardium is exposed to cycles of ischemia and reperfusion; however, the timing of the treatment varies. In a rat I/R model established by Kin et al., three postconditioning cycles were performed at the onset of reperfusion. Each cycle consisted of 10 s reperfusion followed by 10 s reocclusion. This postconditioning protocol decreased I/R-induced damage but demonstrated less cardioprotection as compared to preconditioning, which consisted of 5 min ischemia/10 min reperfusion cycles before the initiation of occlusion [58]. Although the extent of postconditioning in attenuating reperfusion injury remains elusive [59], it is clear that the duration of reperfusion-ischemia cycles and the number of cycles greatly influence the degree of the protective effect [57]. For instance, in a 30 min occlusion model, rats that were treated with three cycles of 30 s reperfusion and ischemia had less infarct size. However, detrimental effects occurred when the duration of treatment in each cycle was lowered to 5 or 15 s [60]. The protective effect of postconditioning has demonstrated a similar mechanism to preconditioning, in which ROS are readily involved. By applying mitoK_ATP_ channel blockers, PKC inhibitors, or ROS scavengers during reperfusion, Penna et al. discovered a unique PKC-oriented redox pathway involved in postconditioning protections [61]. ROS generation during early reperfusion was found to play an essential role in initiating the protective cascade, possibly via the activation of mitoK_ATP_. The mitoK_ATP_ opening raises the level of H_2_O_2_, which ultimately leads to mPTP inhibition and thus prevents cell apoptosis (Figure 1) [57]. It has also been revealed by Hausenloy et al. that the mK_ATP_/ROS/PKC pathways involved in IPC are required to be activated during early reperfusion in order to achieve the protective effect [62]. This is consistent with Downey's finding that IPC exerts protection by triggering the ROS signaling pathway during the initial stage of reperfusion [63]. The emphasis of the redox signaling that occurred at the onset of reperfusion implies a potentially parallel mechanism underlying both IPC and postconditioning. Currently, the effect of postconditioning against I/R has yielded variable results and further research is necessary to evaluate its protection on the heart.

## 5. Pharmacological Strategies in Myocardial I/R Injury

### 5.1. Antioxidants Treatment

Despite the beneficial role of ROS in preconditioning [19], excess ROS have been implicated in the pathogenesis of I/R injury and studies have presented substantial interests in developing therapies to prevent ROS accumulation [7]. In particular, site-targeted treatments such as inhibiting ROS generation at mitochondrial or Nox source may improve the protective effect on the stressed myocardium [19, 64]. Furthermore, the combination of different antioxidants has been found effective in resisting I/R injuries (Figure 1) [19]. Gao et al. have reported that glutathione provides better cardioprotection than ascorbic acid when treated at the beginning of reperfusion in a rat heart model. Moreover, the coadministration of both antioxidants enhances the protective effect as compared to individual treatment [65]. One study examined the effect of VitaePro, a mixed antioxidant compound, and vitamin E in a 21-day oral treatment on rats before the induction of I/R. The results showed that both VitaePro and vitamin E exert cardioprotective effects during I/R, while VitaePro demonstrated a much stronger effect. The work suggests potential prospects of antioxidant drugs in resisting I/R injury [66]. However, other studies on the effectiveness of antioxidant treatments in attenuating I/R-induced damage have yielded varied results [17]. For instance, Flaherty et al. administrated human superoxide dismutase (h-SOD) intravenously to 61 patients prior to coronary angioplasty surgery. Such treatments have failed to demonstrate any improvement on heart function as compared to control group [67]. In fact, several clinical studies have been performed to investigate the role of antioxidant in ROS-mediated reperfusion injuries by administrating antioxidant drugs either before percutaneous coronary intervention or after thrombolysis. Unfortunately, results are not optimistic from the antioxidant interventions in terms of reducing infarct size or enhancing heart function [68].

Apart from antioxidant treatments that scavenge excess ROS, targeting inhibition of ROS production at their own sources may be more favorable. With the sole function of generating ROS in both physiology and disease states [40], Nox has been considered as a therapeutic target in ROS-related injury. Discussed earlier, Nox contributes to a portion of ROS production during reperfusion [26]. In response to I/R injury, both Nox2 and Nox4 isoforms are upregulated in the heart [23]. Although it is reasonable to inhibit Nox activities in order to lessen the ROS activation during I/R injury, a complete inhibition of Nox family is not favorable since Nox is also responsible for the physiological production of ROS. Indeed, a minimal amount of ROS is essential to prevent I/R injury via metabolic adaptations involving hypoxia-inducible factor-1*α* (HIF-1*α*) and peroxisome proliferator-activated receptor-*α*- (PPAR*α*-) dependent mechanisms [23]. Therefore, selective blockage of Nox is highly desirable [23, 40]. Unfortunately, commonly known Nox inhibitors such as apocynin (a Nox2 inhibitor) and diphenyleneiodonium have failed to achieve sufficient specificity [23]. In addition to Nox, accumulated evidence also suggests the involvement of XO in myocardial I/R oxidative injury [69]. Studies evaluating allopurinol, a potent XO inhibitor, have generated positive results on reducing ROS generation and inhibiting cardiomyocyte apoptosis in myocardial infarction models (Figure 1) [70, 71].

Other pharmacologic agents can be used to stimulate conditioning pathways, in order to be as effective as preconditioning treatment. For instance, adenosine reduces myocardial infarct size by activating cardiomyocyte receptors and subsequent PKC pathways that are involved in preconditioning-induced cardioprotection [72]. Cyclosporin administration before or at the onset of reperfusion also significantly ameliorates I/R injury by inhibiting mPTP opening and reserving mitochondrial function [26, 72]. Furthermore, drugs targeting the activation of K_ATP_ channels, such as nicorandil and pioglitazone, have been shown to demonstrate prominent cardioprotection against I/R-induced injuries (Figure 1) [73]. Therefore, activating specific conditioning cascade sites by corresponding drugs highlighted potential treatments to achieve similar protective effect as preconditioning.

### 5.2. Mesenchymal Stem Cell- (MSC-) Based Treatment

Recently, studies have attempted to excavate novel possibilities for treating I/R injuries. Arslan et al. identified that I/R-induced cellular damage may be attributed to the damage of functional proteins, which are essential for fatty acid oxidation, tricarboxylic acid (TCA) cycles, and glycolysis [38]. MSC-derived exosomes can function as reservoirs of functional proteins and alter key biomedical markers of reperfusion injury via paracrine signaling, such as OS, ATP/NADH, and cell death in a positive manner. MSC engraftment also provides reparative effects on tissues that are irreversibly damaged by I/R [38, 74]. Indeed, the injection of MSCs into infarcted myocardium of rats one week after I/R has demonstrated significant improvement in heart conditions including decreased infarct size, indicating the potential therapeutic approach for I/R-induced injury via tissue regeneration [75]. Other research has noticed the potential role of MSCs in suppressing OS and inflammation (Figure 1). Chen et al. have found that I/R-induced ROS production can be limited by injecting adipose-derived MSCs into the rats prior to I/R treatment, which is accompanied by a lower expression of inflammatory and apoptotic biomarkers [14]. All those findings suggest the multiple potentials of MSCs in developing therapeutic interventions to reduce myocardial I/R injuries.

## 6. Conclusions

The current review updated the positive and negative role of ROS in myocardial I/R injury. ROS are responsible for the myocardial damage during both ischemia and reperfusion. On the other hand, a small amount of ROS is essential for exerting cardioprotective effects in preconditioning. Besides IPC, potential treatments such as postconditioning and exercise preconditioning have shown promising results in reducing I/R injury. In addition, specific pharmacological strategies including antioxidants, Nox and XO inhibitors, and MSCs-based treatments are encouraging therapeutics targeting ROS-related I/R injury. Although various therapies have been proposed to prevent or reduce I/R injuries, further research is required to determine their applications in the clinical setting.
